# Supplementary material for: PAbFold: Linear Antibody Epitope Prediction using AlphaFold2
Source: bioRxiv. 2024 Dec 20:2024.04.19.590298. Originally published 2024 Apr 19. Preprint. [Version 2] doi: 10.1101/2024.04.19.590298 (PMC11042291; doi:10.1101/2024.04.19.590298)
Supplement: Supplement 1 [file NIHPP2024.04.19.590298v2-supplement-1.pdf]

bioRxiv preprint doi: https://doi.org/10.1101/2024.04.19.590298; this version posted December 20, 2024. The copyright holder for this preprint (which was not certified by peer review) is the author/funder, who has granted bioRxiv a license to display the preprint in perpetuity. It is made available under aCC-BY 4.0 International license.

Supporting Information

Contents:

Table 1A: Full sequence information for all scFv and antigen proteins
Table 1B: MSA for the scFv chimera variants, with loop and linker region annotation
Figure 1: Comparison of AlphaFold2 Myc scFv predictions to Fab crystal structure
Figure 2: AlphaFold2 predictions for scFv interacting with full length antigen proteins
Figure 3: Illustration of AlphaFold2 peptide predicted placements and confidence thereof
Figure 4: Structure superposition analysis for Myc and HA scFv variants relative to reference crystal structures
Figure 5: In the context of Myc, testing prediction performance versus sliding peptide window parameters
Figure 6: Testing detection of Myc epitope inserted into three locations in an unrelated 3rd-party protein
Figure 7: HA epitope prediction for three anti-HA scFvs
Figure 8: Comparing prediction performance for mBG17 using multimer-v2 and multimer-v3
Figure 9: Comparing prediction performance for Myc using multimer-v2 and multimer-v3 and the new MSA
Figure 10: Comparing prediction performance for HA using multimer-v2 and multimer-v3 and the new MSA
Figure 11: Comparison of 9 major systems after recreating MSAs locally with downloaded databases
Figure 12: Comparison of 9 major systems after recreating MSAs with colabfold after MMSEQS rebuilt the old databases for our use
Figure 13: Comparison of the 9 major systems without using any MSA, using only the single sequence
Figure 14: Comparison of the contents of the MSA for Myc-2E2 after being generated by the 4 major methods: old generation, new generation, local generation, and MMSEQS rebuilt specialty server.
Figure 15: Overview table of whether or not each MSA generation type could accurately detect the experimentally determined epitope in each of the 9 major systems.

bioRxiv preprint doi: https://doi.org/10.1101/2024.04.19.590298; this version posted December 20, 2024. The copyright holder for this preprint (which was not certified by peer review) is the author/funder, who has granted bioRxiv a license to display the preprint in perpetuity. It is made available under aCC-BY 4.0 International license.

Supplemental Table 1A

>mBG17 scFv
MAEVKLEESGGGLVQPGGSMKFSCVASGFTFSDYWMNWVRQSPDKGLEWVAEIRLKSNNYATHYAASVKGRFTISRDDSK
SSVYLQMNNLRAEDSGIYYCTRSAMDYWGQGTSVTVSSGGGGSGGGGSGGGGSDIVMSQSPSSLAVSVGEKITMSCKSS
QSLLYTSDQKNYLAWFQQKPGQSPKLLIFWASTRDSGVPDRFTGSGSGTDFTLTISSVKAEDLAVYYCQQFYNYPRTFGGGT
KLEI

>mBG17-15F11
MAEVKLVESGGGLVKPGGSLKLSCAASGFTFSDYWMNWVRQTPEKRLEWVAEIRLKSNNYATHYAASVKGRFTISRDNAK
NTLYLQMSSLRSEDTAIYYCARSAMDYWGQGTTLTVSSGGGGSGGGGSGGGGSDIVLTQSPASLTVSLGQRATISCKSSQSLL
YTSDQKNYLAWYQQKPGQPPKLLIYWASTRDSGIPARFSGSGSGTDFTLNIHPVEEEDAATYYCQQFYNYPRTFGAGTKLEI

>mBG17-2E2
MAEVQLVESGGDLVKPGGSLKLSCAASGFTFSDYWMNWVRQTPDKRLEWVAEIRLKSNNYATHYAASVKGRFTISRDNAK
NTLYLQMSSLKSEDTAMYYCARSAMDYWGQGTSVTVSSGGGGSGGGGSGGGGSDIVLTQSPASLAVSLGQRATISCKSSQS
LLYTSDQKNYLAWYQQKPGQPPKLLIYWASTRDSGIPARFSGSGSGTDFTLNIHPVEEEDAATYYCQQFYNYPRTFGGGTKLE
I

>mBG17 Fab VH:VL
MYLGLNCVFIVFLLKGVQSEVKLEESGGGLVQPGGSMKFSCVASGFTFSDYWMNWVRQSPDKGLEWVAEIRLKSNNYATH
YAASVKGRFTISRDDSKSSVYLQMNNLRAEDSGIYYCTRSAMDYWGQGTSVTVSS:MDSQAQVLMLLLLWVSGTCGDIVM
SQSPSSLAVSVGEKITMSCKSSQSLLYTSDQKNYLAWFQQKPGQSPKLLIFWASTRDSGVPDRFTGSGS

>mBG17 epitope
DDFSKQLQQS

>mBG17 target protein sequence – SARS CoV-2 Nucleocapsid protein
MSDNGPQNQRNAPRITFGGPSDSTGSNQNGERSGARSKQRRPQGLPNNTASWFTALTQHGKEDLKFPRGQGVPINTNSS
PDDQIGYYRRATRRIRGGDGKMKDLSPRWYFYYLGTGPEAGLPYGANKDGIIWVATEGALNTPKDHIGTRNPANNAAIVLQ
LPQGTTLPKGFYAEGSRGGSQASSRSSSRSRNSSRNSTPGSSRGTSPARMAGNGGDAALALLLLDRLNQLESKMSGKGQQ
QQGQTVTKKSAAEASKKPRQKRTATKAYNVTQAFGRRGPEQTQGNFGDQELIRQGTDYKHWPQIAQFAPSASAFFGMSRI
GMEVTPSGTWLTYTGAIKLDDKDPNFKDQVILLNKHIDAYKTFPPTEPKKDKKKKADETQALPQRQKKQQTVTLLPAADLDD
FSKQLQQSMSSADSTQA

>HA scFv
MAEVKLVESGGDLVKPGGSLKLSCAASGFTFSSYGMSWVRQTPDKRLEWVATISRGGSYTYYPDSVKGRFTISRDNAKNTLY
LQMSSLKSEDTAMYYCARRETYDEKGFAYWGQGTTVTVSSGGGGSGGGGSGGGGSDIELTQSPSSLTVTAGEKVTMSCKSS
QSLLNSGNQKNYLTWYQQKPGQPPKLLIYWASTRESGVPDRFTGSGSGRDFTLTISSVQAEDLAVYYCQNDNSHPLTFGAG
TKLEL

>HA-15F11

bioRxiv preprint doi: https://doi.org/10.1101/2024.04.19.590298; this version posted December 20, 2024. The copyright holder for this preprint (which was not certified by peer review) is the author/funder, who has granted bioRxiv a license to display the preprint in perpetuity. It is made available under aCC-BY 4.0 International license.

MEVKLVESGGGLVKPGGSLKLSCAASGFTFSSYGMSWVRQTPEKRLEWVATISRGGSYTYYPDSVKGRFTISRDNAKNTLYL
QMSSLRSEDTAIYYCARRETYDEKGFAYWGQGTTLTVSSGGGGSGGGGSGGGGSDIVLTQSPASLTVSLGQRATISCKSSQSL
LNSGNQKNYLTWYQQKPGQPPKLLIYWASTRESGIPARFSGSGSGTDFTLNIHPVEEEDAATYYCQNDNSHPLTFGAGTKLEI

>HA-2E2
MAEVQLVESGGDLVKPGGSLKLSCAASGFTFSSYGMSWVRQTPDKRLEWVATISRGGSYTYYPDSVKGRFTISRDNAKNTLY
LQMSSLKSEDTAMYYCARRETYDEKGFAYWGQGTSVTVSSGGGGSGGGGSGGGGSDIVLTQSPASLAVSLGQRATISCKSS
QSLLNSGNQKNYLTWYQQKPGQPPKLLIYWASTRESGIPARFSGSGSGTDFTLNIHPVEEEDAATYYCQNDNSHPLTFGGGT
KLEI

>HA target protein sequence – influenza hemmaglutanin A
MKTIIALSYILCLVSAQKLPGSENRTATLCLGHHAVQNGTLVKTITNDQIEVTNATELVQSSSTGRICDNPHRVLDGRDCTLIDA
LLGDPHCDSFQNKEWDLFIERSKAYSNCYPYDVPDYASLRSLVASSGTLEFTTEGFDWTGVTQNGTSYSCKRGSANSFFSRLN
WLHKLNYKYPAQNVTMPNDDKFDKLYIWGVHHPSTDNDQTSLYVQTSGRVTVSTKRSQQTVVPDIGSRPWVRGISSRISIH
WTIVKPGDILLINSTGNLIAPRGYFKIRNGKSSIMKSDALIGNCNSECITPNGSIPNDKPFQNVNRITYGDCPRYVKQSTLKLAT
GMRNVPEKQTRGIFGAIAGFIENGWEGMVDGWYGFRHRNSEGTGQAADLKSTQAAIDQINGKLNRLIKKTNEKFHQIEKE
FSEVEGRIQDLEKYVEDTKVDLWSYNAELLVALENQHTIDLTDSEMNKLFERTRKQLRENAEDMGNGCFKIYHRCDNACIGS
IRNGTYNHNVYRDEALNNRFKIKGVELKSGYKDWILWISFAISCFLLCVGLMGLIMWTCQKGNIRCIRCNICH

>HA epitope
YPYDVPDYA

>Myc scFv
MEVKLVESGGDLVKPGGSLKLSCAASGFTFSHYGMSWVRQTPDKRLEWVATIGSRGTYTHYPDSVKGRFTISRDNDKNALY
LQMNSLKSEDTAMYYCARRSEFYYYGNTYYYSAMDYWGQGASVTVSSGGGGSGGGGSGGGGSDIVLTQSPASLAVSLGQ
RATISCRASESVDNYGFSFMNWFQQKPGQPPKLLIYAISNRGSGVPARFSGSGSGTDFSLNIHPVEEDDPAMYFCQQTKEVP
WTFGGGTKLEI

>Myc-15F11
MEVKLVESGGGLVKPGGSLKLSCAASGFTFSHYGMSWVRQTPEKRLEWVATIGSRGTYTHYPDSVKGRFTISRDNAKNTLYL
QMSSLRSEDTAIYYCARRSEFYYYGNTYYYSAMDYWGQGTTLTVSSGGGGSGGGGSGGGGSDIVLTQSPASLTVSLGQRATI
SCRASESVDNYGFSFMNWYQQKPGQPPKLLIYAISNRGSGIPARFSGSGSGTDFTLNIHPVEEEDAATYYCQQTKEVPWTFG
AGTKLEI

>Myc-2E2
MAEVQLVESGGDLVKPGGSLKLSCAASGFTFSHYGMSWVRQTPDKRLEWVATIGSRGTYTHYPDSVKGRFTISRDNAKNTL
YLQMSSLKSEDTAMYYCARRSEFYYYGNTYYYSAMDYWGQGTSVTVSSGGGGSGGGGSGGGGSDIVLTQSPASLAVSLGQ
RATISCRASESVDNYGFSFMNWYQQKPGQPPKLLIYAISNRGSGIPARFSGSGSGTDFTLNIHPVEEEDAATYYCQQTKEVP
WTFGGGTKLEI

>Myc target protein sequence
MDFFRVVENQPPATMPLNVSFTNRNYDLDYDSVQPYFYCDEEENFYQQQQQSELQPPAPSEDIWKKFELLPTPPLSPSRRS
GLCSPSYVAVTPFSLRGDNDGGGGSFSTADQLEMVTELLGGDMVNQSFICDPDDETFIKNIIIQDCMWSGFSAAAKLVSEKL
ASYQAARKDSGSPNPARGHSVCSTSSLYLQDLSAAASECIDPSVVFPYPLNDSSSPKSCASQDSSAFSPSSDSLLSSTESSPQGS

bioRxiv preprint doi: https://doi.org/10.1101/2024.04.19.590298; this version posted December 20, 2024. The copyright holder for this preprint (which was not certified by peer review) is the author/funder, who has granted bioRxiv a license to display the preprint in perpetuity. It is made available under aCC-BY 4.0 International license.

PEPLVLHEETPPTTSSDSEEEQEDEEEIDVVSVEKRQAPGKRSESGSPSAGGHSKPPHSPLVLKRCHVSTHQHNYAAPPSTRK
DYPAAKRVKLDSVRVLRQISNNRKCTSPRSSDTEENVKRRTHNVLERQRRNELKRSFFALRDQIPELENNEKAPKVVILKKATA
YILSVQAEEQKLISEEDLLRKRREQLKHKLEQLRNSCA

>Myc epitope
EQKLISEEDL

Supplemental Table 1B

bioRxiv preprint doi: https://doi.org/10.1101/2024.04.19.590298; this version posted December 20, 2024. The copyright holder for this preprint (which was not certified by peer review) is the author/funder, who has granted bioRxiv a license to display the preprint in perpetuity. It is made available under aCC-BY 4.0 International license.

```
Kabat numbering      1--------10--------20--------30--------40--------50-------
MYC                 -MEVKLVESGGDLVKPGGSLKLSCAASGFTFSHYGMSWVRQTPDKRLEWVATIG--SRGT
MYC-2E2             MAEVQLVESGGDLVKPGGSLKLSCAASGFTFSHYGMSWVRQTPDKRLEWVATIG--SRGT
MYC-15F11           -MEVKLVESGGGLVKPGGSLKLSCAASGFTFSHYGMSWVRQTPEKRLEWVATIG--SRGT
mBG17               MAEVKLEESGGGLVQPGGSMKFSCVASGFTFSDYWMNWVRQSPDKGLEWVAEIRLKSNNY
mBG17-2E2           MAEVQLVESGGDLVKPGGSLKLSCAASGFTFSDYWMNWVRQTPDKRLEWVAEIRLKSNNY
mBG17-15F11         MAEVKLVESGGGLVKPGGSLKLSCAASGFTFSDYWMNWVRQTPEKRLEWVAEIRLKSNNY
HA-scFv             MAEVKLVESGGDLVKPGGSLKLSCAASGFTFSSYGMSWVRQTPDKRLEWVATISRG--GS
HA-2E2              MAEVQLVESGGDLVKPGGSLKLSCAASGFTFSSYGMSWVRQTPDKRLEWVATISRG--GS
HA-15F11            -MEVKLVESGGGLVKPGGSLKLSCAASGFTFSSYGMSWVRQTPEKRLEWVATISRG--GS

Kabat numbering     ---------65---70--------80-----------90---95--------------102
MYC                 YTHYPDSVKGRFTISRDNDKNALYLQMNSLKSEDTAMYYCARRSEFYYYGNTYYYSAMDY
MYC-2E2             YTHYPDSVKGRFTISRDNAKNTLYLQMSSLKSEDTAMYYCARRSEFYYYGNTYYYSAMDY
MYC-15F11           YTHYPDSVKGRFTISRDNAKNTLYLQMSSLRSEDTAIYYCARRSEFYYYGNTYYYSAMDY
mBG17               ATHYAASVKGRFTISRDDSKSSVYLQMNNLRAEDSGIYYCTRS-------------AMDY
mBG17-2E2           ATHYAASVKGRFTISRDNAKNTLYLQMSSLKSEDTAMYYCARS-------------AMDY
mBG17-15F11         ATHYAASVKGRFTISRDNAKNTLYLQMSSLRSEDTAIYYCARS-------------AMDY
HA-scFv             YTYYPDSVKGRFTISRDNAKNTLYLQMSSLKSEDTAMYYCARRET-------YDEKGFAY
HA-2E2              YTYYPDSVKGRFTISRDNAKNTLYLQMSSLKSEDTAMYYCARRET-------YDEKGFAY
HA-15F11            YTYYPDSVKGRFTISRDNAKNTLYLQMSSLRSEDTAIYYCARRET-------YDEKGFAY

                    -------110-               1--------10------------24---------
MYC                 WGQGASVTVSSGGGGSGGGGSGGGGSDIVLTQSPASLAVSLGQRATISCRASESVDNYG-
MYC-2E2             WGQGTSVTVSSGGGGSGGGGSGGGGSDIVLTQSPASLAVSLGQRATISCRASESVDNYG-
MYC-15F11           WGQGTTLTVSSGGGGSGGGGSGGGGSDIVLTQSPASLTVSLGQRATISCRASESVDNYG-
mBG17               WGQGTSVTVSSGGGGSGGGGSGGGGSDIVMSQSPSSLAVSVGEKITMSCKSSQSLLYTSD
mBG17-2E2           WGQGTSVTVSSGGGGSGGGGSGGGGSDIVLTQSPASLAVSLGQRATISCKSSQSLLYTSD
mBG17-15F11         WGQGTTLTVSSGGGGSGGGGSGGGGSDIVLTQSPASLTVSLGQRATISCKSSQSLLYTSD
HA-scFv             WGQGTTVTVSSGGGGSGGGGSGGGGSDIELTQSPSSLTVTAGEKVTMSCKSSQSLLNSGN
HA-2E2              WGQGTSVTVSSGGGGSGGGGSGGGGSDIVLTQSPASLAVSLGQRATISCKSSQSLLNSGN
HA-15F11            WGQGTTLTVSSGGGGSGGGGSGGGGSDIVLTQSPASLTVSLGQRATISCKSSQSLLNSGN

                    -----34----40--------50--------60--------70--------80-------
MYC                 -FSFMNWFQQKPGQPPKLLIYAISNRGSGVPARFSGSGSGTDFSLNIHPVEEDDPAMYFC
MYC-2E2             -FSFMNWYQQKPGQPPKLLIYAISNRGSGIPARFSGSGSGTDFTLNIHPVEEEDAATYYC
MYC-15F11           -FSFMNWYQQKPGQPPKLLIYAISNRGSGIPARFSGSGSGTDFTLNIHPVEEEDAATYYC
mBG17               QKNYLAWFQQKPGQSPKLLIFWASTRDSGVPDRFTGSGSGTDFTLTISSVKAEDLAVYYC
mBG17-2E2           QKNYLAWYQQKPGQPPKLLIYWASTRDSGIPARFSGSGSGTDFTLNIHPVEEEDAATYYC
mBG17-15F11         QKNYLAWYQQKPGQPPKLLIYWASTRDSGIPARFSGSGSGTDFTLNIHPVEEEDAATYYC
HA-scFv             QKNYLTWYQQKPGQPPKLLIYWASTRESGVPDRFTGSGSGRDFTLTISSVQAEDLAVYYC
HA-2E2              QKNYLTWYQQKPGQPPKLLIYWASTRESGIPARFSGSGSGTDFTLNIHPVEEEDAATYYC
HA-15F11            QKNYLTWYQQKPGQPPKLLIYWASTRESGIPARFSGSGSGTDFTLNIHPVEEEDAATYYC

                    -90--------100----
MYC                 QQTKEVPWTFGGGTKLEI
MYC-2E2             QQTKEVPWTFGGGTKLEI
MYC-15F11           QQTKEVPWTFGAGTKLEI
mBG17               QQFYNYPRTFGGGTKLEI
mBG17-2E2           QQFYNYPRTFGGGTKLEI
mBG17-15F11         QQFYNYPRTFGAGTKLEI
HA-scFv             QNDNSHPLTFGAGTKLEL
HA-2E2              QNDNSHPLTFGGGTKLEI
HA-15F11            QNDNSHPLTFGAGTKLEI
```

Legend: Heavy chain loops | linker | Light chain loops

bioRxiv preprint doi: https://doi.org/10.1101/2024.04.19.590298; this version posted December 20, 2024. The copyright holder for this preprint (which was not certified by peer review) is the author/funder, who has granted bioRxiv a license to display the preprint in perpetuity. It is made available under aCC-BY 4.0 International license.

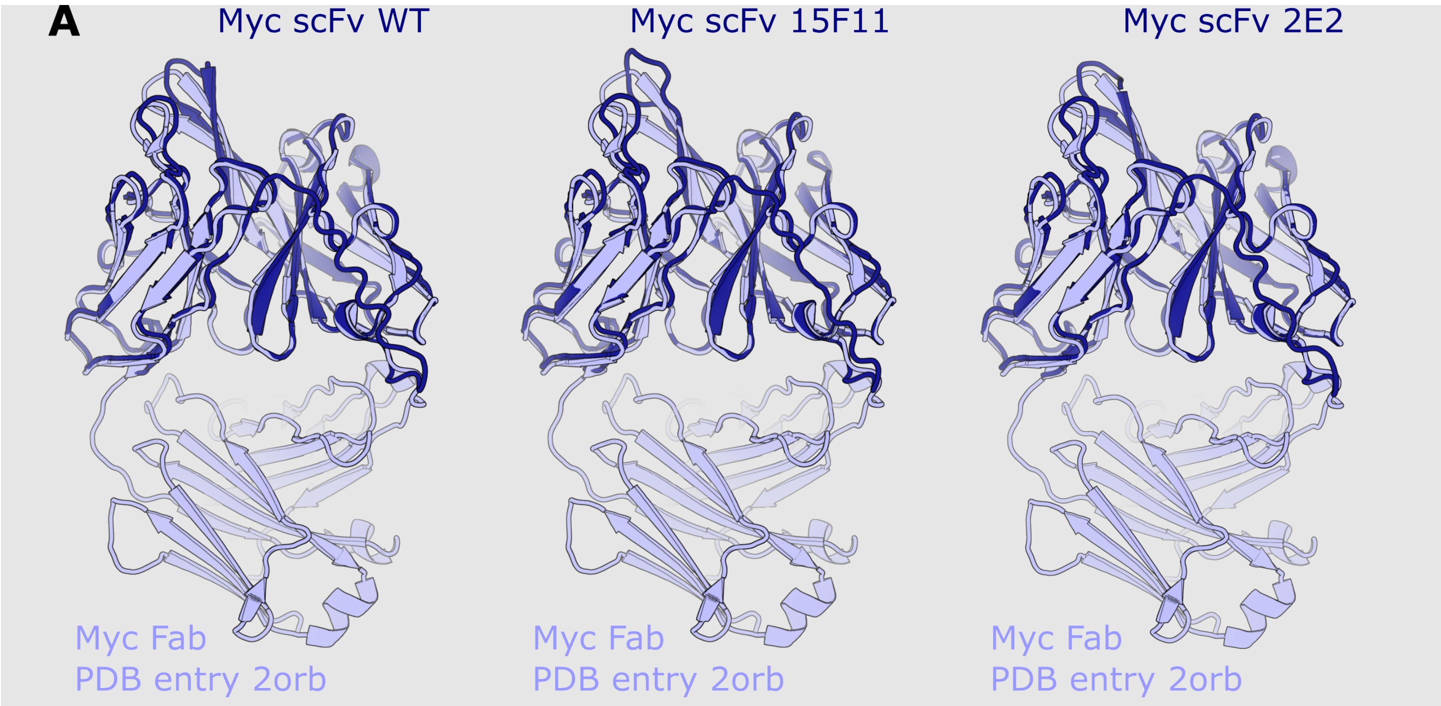

B

Structure	RMSD $_{C\alpha}$ [Å] vs 2orb
Myc scFv WT	0.42
Myc scFv 15F11	0.45
Myc scFv 2E2	0.45

Supplemental Figure 1. Alignment of AlphaFold2 predicted scFv structures to an anti-c-Myc Fab crystal structure. A) Alignments of AlphaFold2-derived wild-type Myc scFv, Myc-2E2 scFv, and Myc-15F11 scFv structures with a Myc Fab crystal structure (PDB: 2orb). Predicted scFv structures are shown in dark blue, 2orb Myc Fab structures are shown in light blue. **B)** RMSD values comparing structural similarities between the wild-type Myc scFv, Myc-2E2 scFv, and Myc-15F11 scFv structures with a Myc Fab crystal structure (PDB: 2orb) were computed by the PyMOL align command.

bioRxiv preprint doi: https://doi.org/10.1101/2024.04.19.590298; this version posted December 20, 2024. The copyright holder for this preprint (which was not certified by peer review) is the author/funder, who has granted bioRxiv a license to display the preprint in perpetuity. It is made available under aCC-BY 4.0 International license.

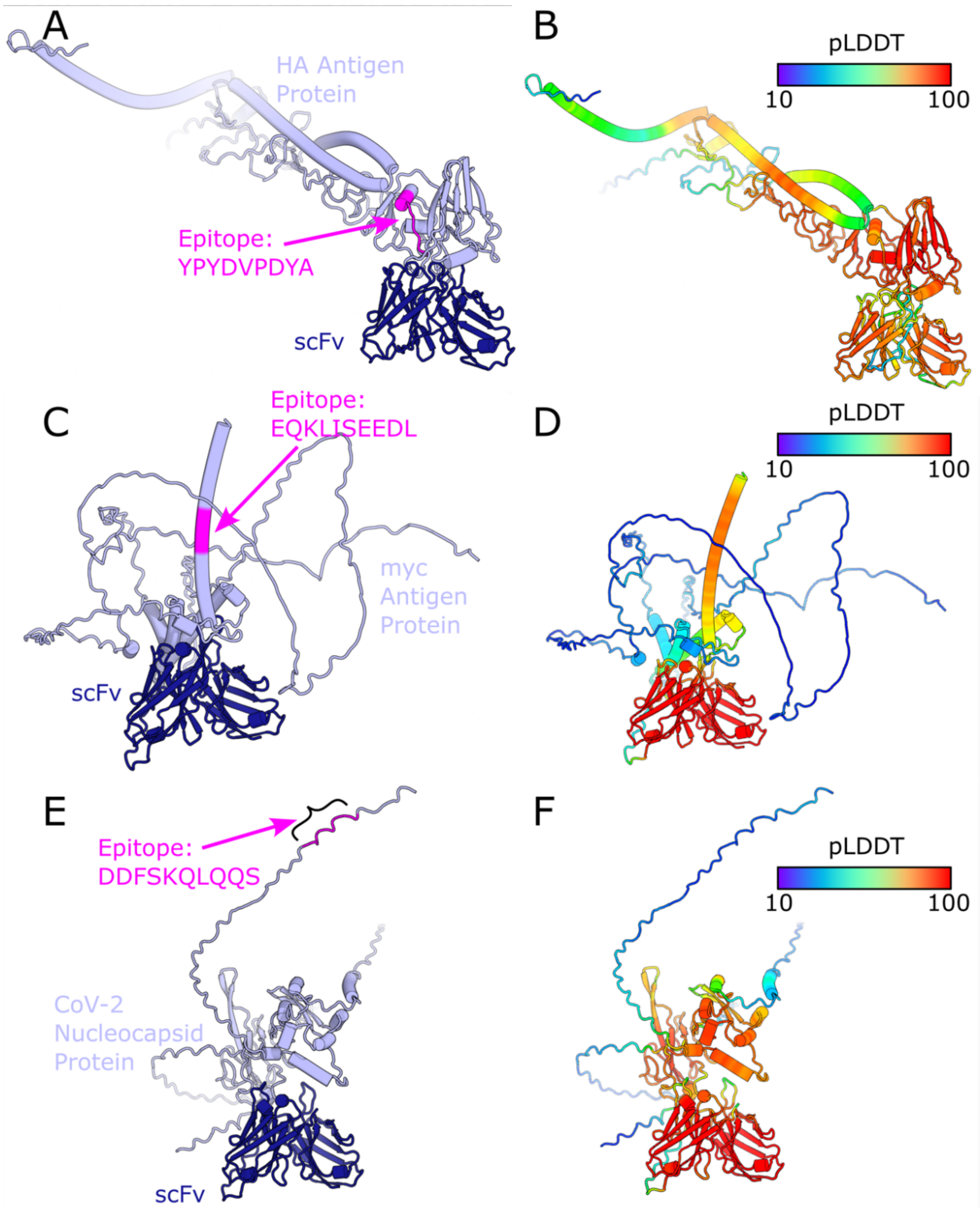

Supplemental Figure 2: Alphafold2's best attempt to dock whole sequences with the respective sequence's scFv. **A)** The whole HA protein structure and scFv complex as predicted by AF2, with the correct epitope sequence highlighted in magenta. **B)** Shows the same structure by highlighted by confidence (pLDDT) of the structure with AF2. Similarly, the entire Myc protein-scFv complex are shown with **C)** the correct epitope highlighted in magenta and **D)** the confidence of the structure shown, and again for the mBG17 N-protein-scFv complex in **E)** and **F)**.

bioRxiv preprint doi: https://doi.org/10.1101/2024.04.19.590298; this version posted December 20, 2024. The copyright holder for this preprint (which was not certified by peer review) is the author/funder, who has granted bioRxiv a license to display the preprint in perpetuity. It is made available under aCC-BY 4.0 International license.

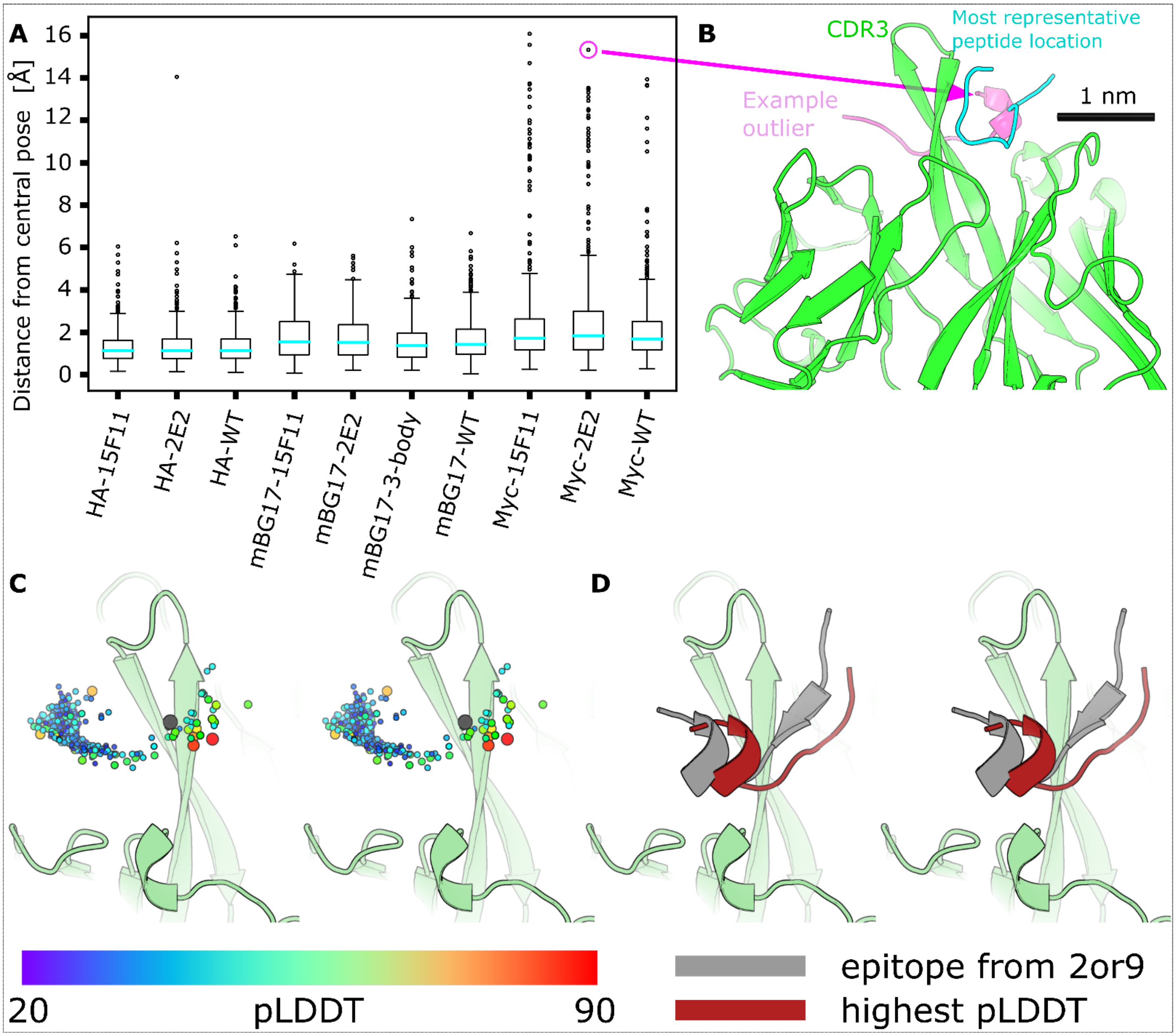

Supplemental Figure 3: AlphaFold2 places all peptides near the CDR loops. The predicted Cα coordinates for all scFv (excluding the flexible linker) were extracted, and all were aligned together using the Kabsch algorithm (48, 49). With the scFvs structurally aligned, an all-against-all RMSD was calculated for the epitope peptides. To visually represent each peptide as a single point, the coordinates for all epitope atoms were averaged. The "central" exemplar epitope (cyan) is the peptide with the smallest sum of RMSD to all other peptides. **A)** The average and quartile for peptide placement relative to the central peptide via Box-and-Whisker plot reveals that AlphaFold2 largely places all epitopes in the same area. The Myc CDRH3 runs through the middle of a traditional paratope pocket, it isn't a "cradle" for the epitope to sit on. AlphaFold2 places peptides on both sides of the CDRH3, causing significant spread in the peptide placement. **B)** An example of an exemplar, most-central predicted peptide structure (cyan) for the peptide PKSCASQDSS (cyan) bound to the Myc-2E2 scFv (green) that is distant from an example outlier peptide (magenta, peptide PHSPLVLKRC, center-to-center distance 14.8 Å). All peptide placements are still in contact with CDRH3, consistent with a strong AlphaFold2 bias to place peptides in a typical antibody binding site. **C)** The Myc-2E2 scFv (pale-green) and the average epitope placement (cyan) peptide alongside the crystal structure solution of the Myc epitope (grey). Remaining peptide placements are represented as a cloud of spheres at the mean peptide position. Each peptide sphere is colored and sized

bioRxiv preprint doi: https://doi.org/10.1101/2024.04.19.590298; this version posted December 20, 2024. The copyright holder for this preprint (which was not certified by peer review) is the author/funder, who has granted bioRxiv a license to display the preprint in perpetuity. It is made available under aCC-BY 4.0 International license.

by epitope pLDDT (ranging from 20 to 90). Although AlphaFold2 frequently placed peptides on the opposite side of the CDRH3 from the Myc epitope (grey), it was not confident in these peptide placements (low, small, blue pLDDT spheres). In contrast, some of the peptides placed around the CDRH3, and in positions similar to the native epitope (grey) were placed with higher pLDDT confidence (increasingly large spheres trending from green to yellow to orange and red). **D)** The top ranked peptide as predicted by PAbFold with sequence QKLISEEDLL (red) and the crystal structure solution of the Myc epitope (grey).

bioRxiv preprint doi: https://doi.org/10.1101/2024.04.19.590298; this version posted December 20, 2024. The copyright holder for this preprint (which was not certified by peer review) is the author/funder, who has granted bioRxiv a license to display the preprint in perpetuity. It is made available under aCC-BY 4.0 International license.

scFv	Apo			Docked		
	BB Ca RMSD	Loop all backbone RMSD	Epitope all atom RMSD	BB Ca RMSD	Loop all backbone RMSD	Epitope all atom RMSD
Myc	0.65	2.87	NA	0.47	1.75	6.69
Myc-15F11	0.62	3.06	NA	0.51	1.51	2.45
Myc-2E2	0.61	2.96	NA	0.51	1.61	2.68

scFv	Apo			Docked		
	BB Ca RMSD	Loop all backbone RMSD	Epitope all atom RMSD	BB Ca RMSD	Loop all backbone RMSD	Epitope all atom RMSD
HA	0.56	1.39	NA	0.58	1.25	3.2
HA-15F11	0.56	1.32	NA	0.6	1.26	3.1
HA-2E2	0.58	1.21	NA	0.6	1.27	3.1

Supplemental Figure 4: RMSD comparison (all numbers have units of Å) for AlphaFold2 predicted scFv structures compared to reference crystal structures, **A)** 2or9 (Myc) and **B)** 1frg (HA), respectively. The loops of the scFv more closely mimic the crystal structure when the epitope peptide is present. The backbone also undergoes subtle changes during docking that make it slightly more similar to the crystal structure. These structures were aligned by identifying the framework residues in all structures, then aligning the framework region Cα with the Kabsch algorithm (48, 49). Specifically excluded from this process were the heavy and light CDR loops of the structures, as well as the flexible linker structure that connects the heavy and light chains due to the inherent floppy, unstructured nature of this region. After aligning the framework regions of the AlphaFold2 predicted structures and the crystal structures (2or9 and 1frg respectively), an RMSD of these Cα was calculated and is reported as the first column 'BB Cα RMSD'. Without further alignment, loop placement was analyzed with an all backbone RMSD by calculating the RMSD between the C, Cα, N, and O along the backbone of all residues in the scFv that were not used for the framework superimposition. This RMSD is reported in the second column as 'Loop all backbone RMSD'. Finally, to investigate peptide predicted placement and potential scFv:epitope interactions, an all-atom RMSD was calculated between the crystal structure and the AF2 predicted peptide structure (no additional alignment). Because the apo structure lacks a peptide position, this is only reported in the 'Docked' category and is in the 3rd column labeled 'Epitope all atom RMSD'. One script was written for each scFv (Myc and HA), and can be found in the Zenodo deposition of our data (https://zenodo.org/records/10884181) because this analysis is not a key part of PAbFold. Briefly this analysis reveals that all three HA scFv variants have predicted framework regions and loop regions in the apo structures that closely match the reference structure (0.56-0.58 Å and 1.21-1.39 Å). Accordingly, when the cognate epitope peptide is present, it can be placed with relatively high accuracy for all three scFvs (3.1-3.2 Å), with only small changes in the loops (1.39 Å to 1.25 Å, 1.32 Å to 1.26 Å, and 1.21 Å to 1.27 Å). In contrast, the apo structures for the three Myc scFvs have a much higher deviation in the loop regions (2.87 to 3.06 Å). When the epitope peptide is added, there is significant motion in the loops consistent with an "induced fit" description. In the two chimeric Myc scFvs (Myc-15F11 and Myc-2E2) the final loop RMSD is reduced to 1.51-1.61 Å, and the epitope peptide is successfully predicted (2.45-2.68 Å). However, despite a lower apo-state loop RMSD (2.87 Å), the loop RMSD for the wild-type Myc scFv only drops to 1.75 Å, and the epitope peptide placement does not match the experimental structure (6.69 Å). This is consistent with the failure of the wild-type Myc scFv AlphaFold2 predictions in main text Figure 2.

bioRxiv preprint doi: https://doi.org/10.1101/2024.04.19.590298; this version posted December 20, 2024. The copyright holder for this preprint (which was not certified by peer review) is the author/funder, who has granted bioRxiv a license to display the preprint in perpetuity. It is made available under aCC-BY 4.0 International license.

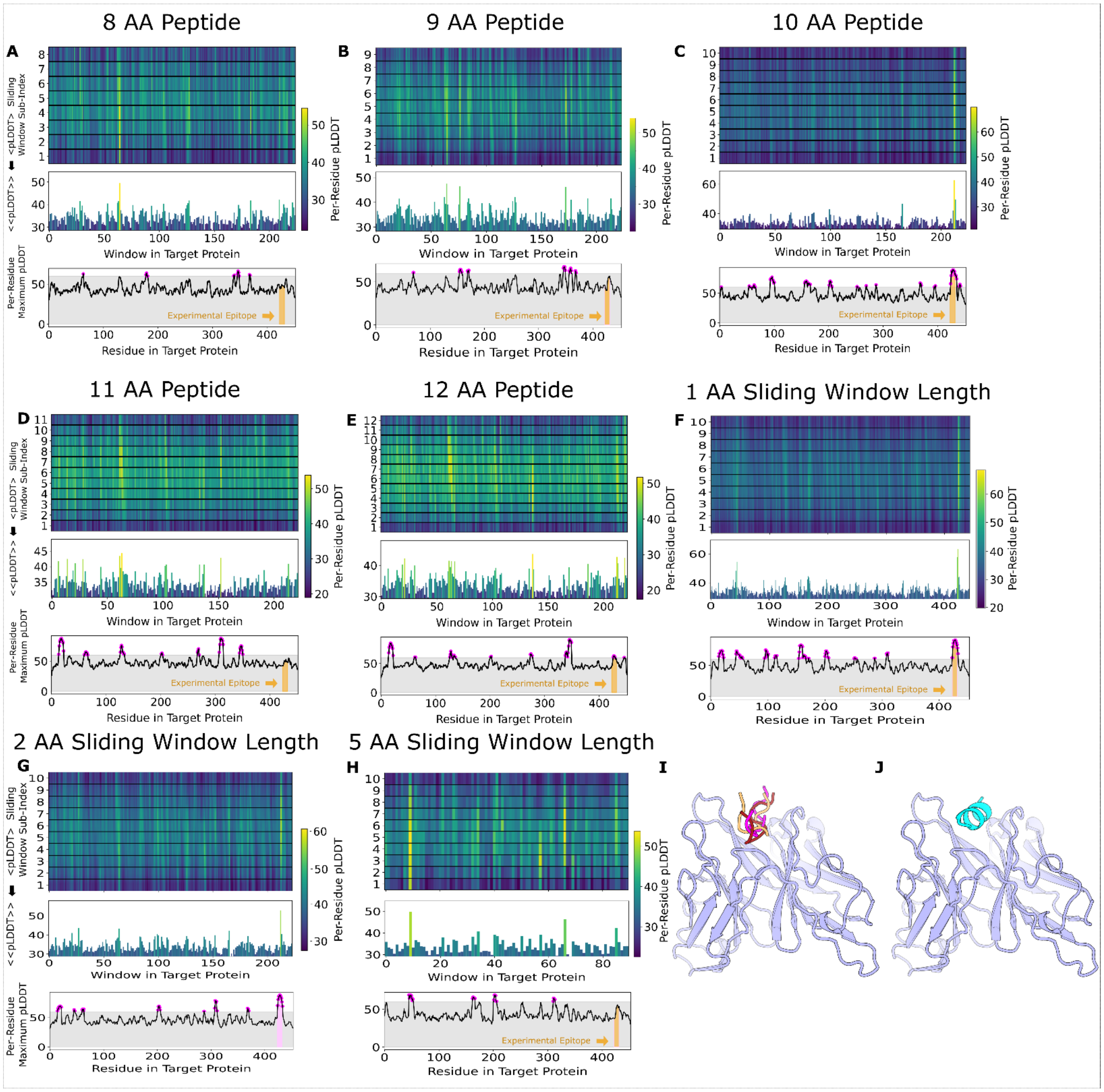

Supplemental Figure 5. Assessment of peptide size and sliding window sizes on epitope prediction efficacy. Myc-2E2 scFv:peptide structures were predicted with peptides of 8 (**A**), 9 (**B**), 10 (**C**), 11 (**D**), and 12 (**E**) amino acid lengths derived from the Myc protein with a sliding window of 2 amino acids, and pLDDT scores from each predicted structure were plotted against the Myc amino acid position and sliding window length target. **F)** Negative control peptides bind to antibody binding sites, but with poor pLDDT scores. Similarly, with a fixed peptide length of 10 and a sliding window step size of 1 (**F**), 2 (**G**), and 5 (**H**), we can see the practical epitope detection outcome was similar for a sliding window of 1 and 2, but resolution and accuracy were reduced for a sliding window step size of 5. To more fully illustrate the strong learned bias that AlphaFold2 has for placing any peptides among the CDR loops, we predicted the structure of Myc-2E2 in complex with several control peptides. These negative control peptides bind to the generally expected antibody binding site, but with poor pLDDT. **I)** GSx5 in magenta (GSGSGSGSGS) had a score (mean peptide from Simple Max method pLDDT) of 29.5. $(GGGGS)_2$ in orange (GGGGSGGGGS) had a score of 31.9. G_{10} in red (GGGGGGGGGG) had a score of 33. Lastly, **J)** A_{10} in cyan (AAAAAAAAAA) had a score of 41 and is the only negative control peptide to have an alpha-helical secondary structure (presumably due to the increased alpha helical propensity of alanine).

bioRxiv preprint doi: https://doi.org/10.1101/2024.04.19.590298; this version posted December 20, 2024. The copyright holder for this preprint (which was not certified by peer review) is the author/funder, who has granted bioRxiv a license to display the preprint in perpetuity. It is made available under aCC-BY 4.0 International license.

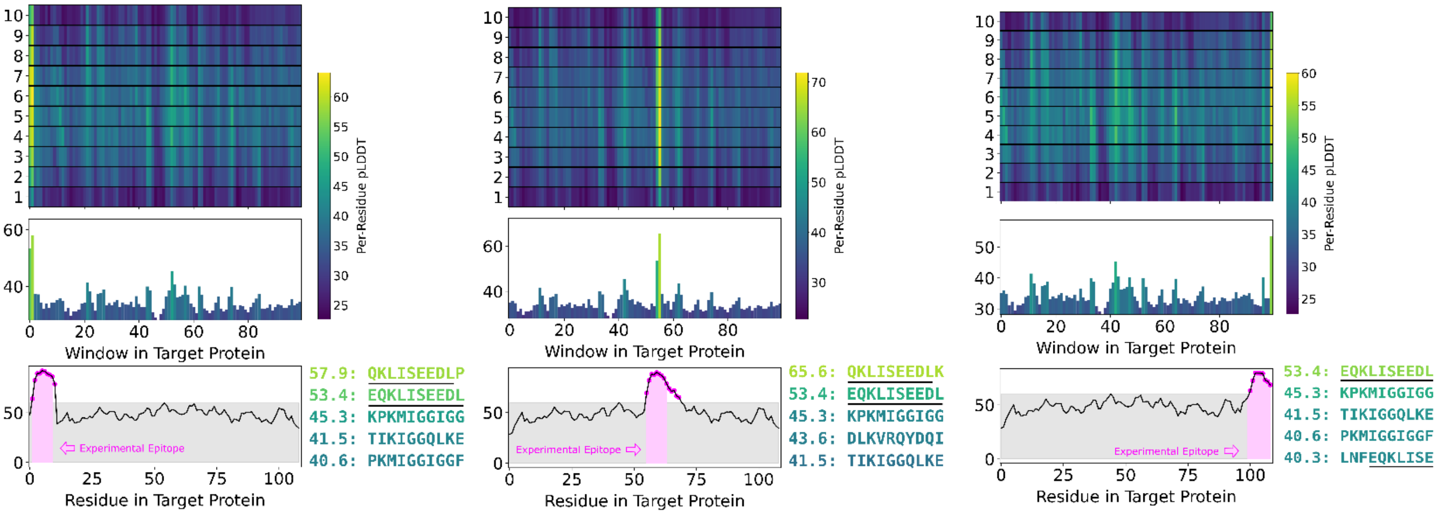

Supplemental Figure 6: PAbFold epitope detection is independent of position within target sequence. The Myc epitope (EQKLISEEDL) was added into the beginning, middle, or end of the 99-a.a. HIV protease sequence (Genbank Accession: NP_705926.1) prior to epitope scanning structure prediction. Positions of the Myc epitope sequence added to in the **A)** N-terminus **B)** middle and **C)** C-terminus of the HIV protease sequence. **D)** Highlights the ranked sequences recovered from each experiment in A, B, and C.

bioRxiv preprint doi: https://doi.org/10.1101/2024.04.19.590298; this version posted December 20, 2024. The copyright holder for this preprint (which was not certified by peer review) is the author/funder, who has granted bioRxiv a license to display the preprint in perpetuity. It is made available under aCC-BY 4.0 International license.

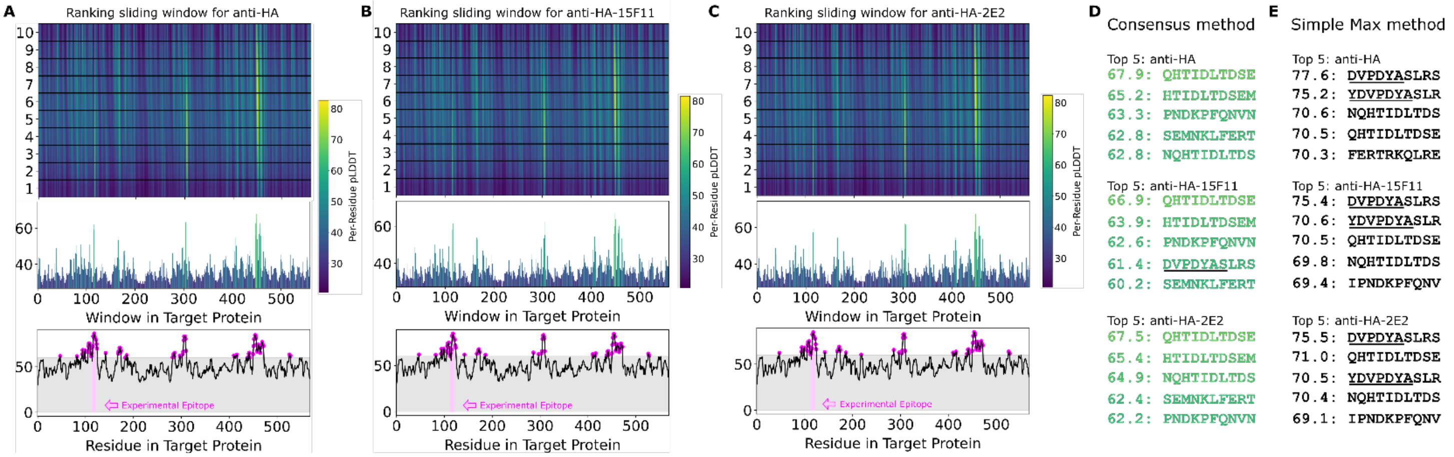

Supplemental Figure 7: Alphafold2 can accurately predict the HA linear epitope in different scFv backbones. The anti-HA VH and VL antibody sequences were used to generate either **A)** wild-type scFv or CDR loop grafted onto the **B)** 15F11 or **C)** 2E2 antibody backbones. The Influenza A virus hemagglutinin protein sequence (Genbank AUT17530.1) was used as the target antigen and processed into 10 amino acid overlapping peptides with a 1 amino acid sliding window. The structures for each scFv:peptide pair were predicted with Alphafold2, and pLDDT values for each scFv:peptide pair are shown. **D)** The top-ranking epitope sequences via pLDDT scores are reported via the consensus method. Sequence underlining represents overlap with the known HA epitope (HA a.a. 114-125: YDVPDYASL). **E)** The top-ranking epitope sequences via pLDDT scores are reported via the simple max method.

bioRxiv preprint doi: https://doi.org/10.1101/2024.04.19.590298; this version posted December 20, 2024. The copyright holder for this preprint (which was not certified by peer review) is the author/funder, who has granted bioRxiv a license to display the preprint in perpetuity. It is made available under aCC-BY 4.0 International license.

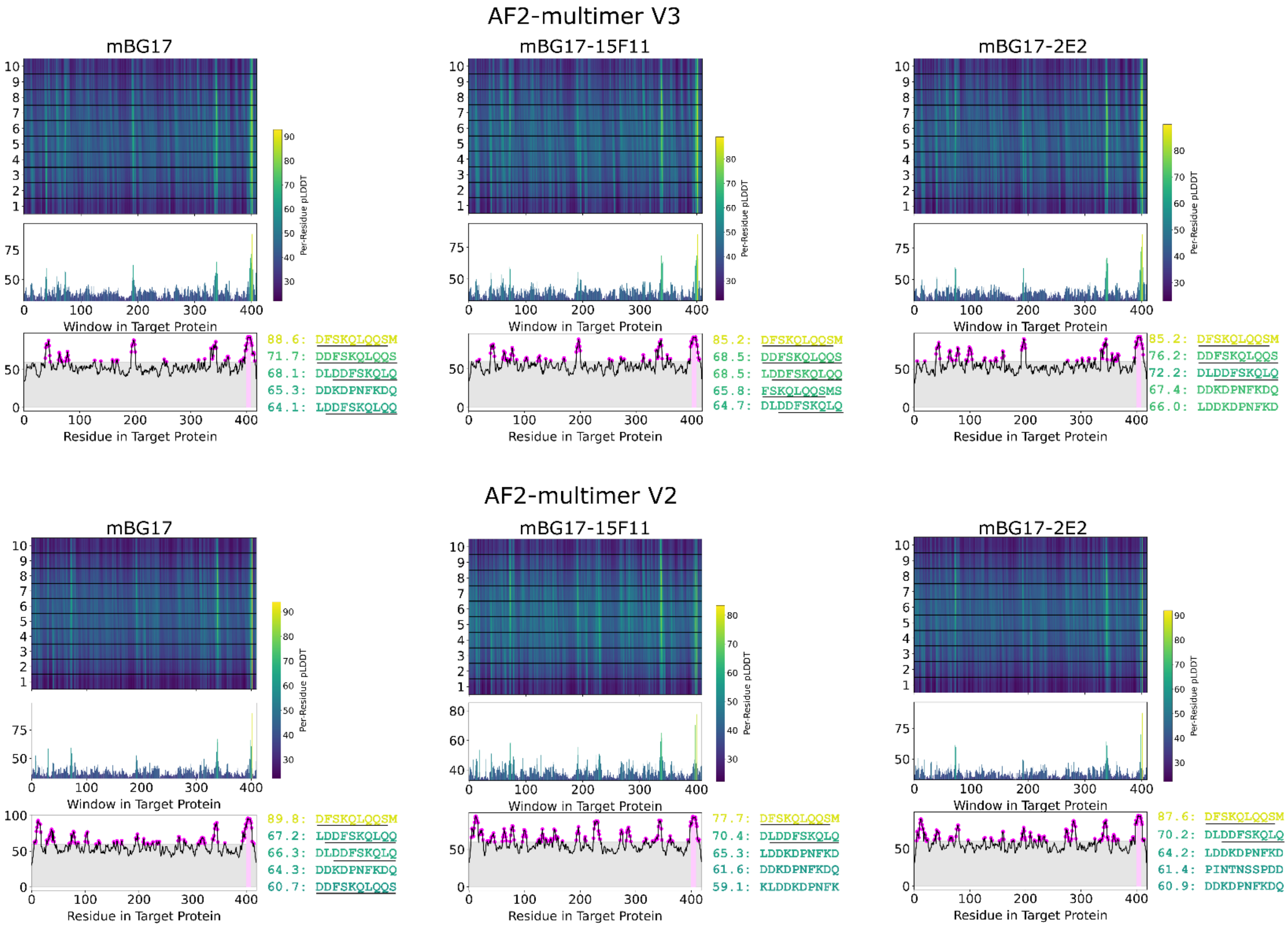

Supplemental Figure 8: A comparison of Alphafold2 multimer version 3 and multimer version 2 applied to the mBG17 system. The experimental epitope, DDFSKQLQQS, is still easily identified with all three scFv backbones (wildtype, 15F11, and 2E2).

bioRxiv preprint doi: https://doi.org/10.1101/2024.04.19.590298; this version posted December 20, 2024. The copyright holder for this preprint (which was not certified by peer review) is the author/funder, who has granted bioRxiv a license to display the preprint in perpetuity. It is made available under aCC-BY 4.0 International license.

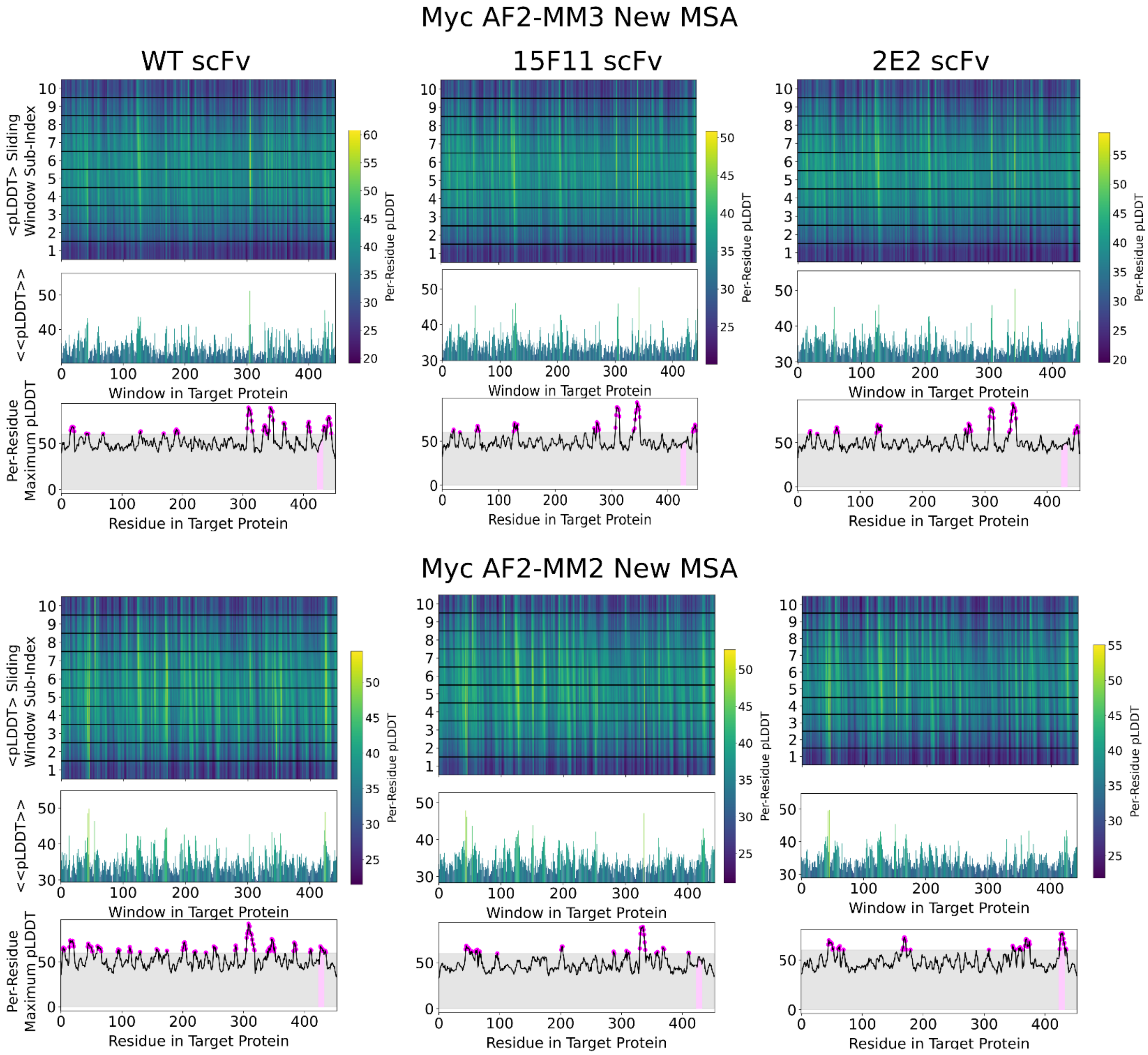

Supplemental Figure 9: Myc comparison of epitope identification accuracy, comparing model types. Performance variation with AlphaFold2 model (multiple versions 2 and 3) and MSA versions (most up to date version of the ColabFold MSA server uses UniRef30 (2302) and PDB100 (220517)) vs the old MSA server (when this data was initially generated, ColabFold MSA server used UniRef30 (2202) and PDB70 (220313)). The left column is the WT scFv, the middle column is the CDR loops spliced onto the 15F11 backbone, and the right column is the CDR loops spliced onto the 2E2 backbone. Performance was ablated when using MM3 and the new MSA, and significantly degraded when using MM2 with the new MSA. For AF2-MM2 Old MSA, see Figure 2.

bioRxiv preprint doi: https://doi.org/10.1101/2024.04.19.590298; this version posted December 20, 2024. The copyright holder for this preprint (which was not certified by peer review) is the author/funder, who has granted bioRxiv a license to display the preprint in perpetuity. It is made available under aCC-BY 4.0 International license.

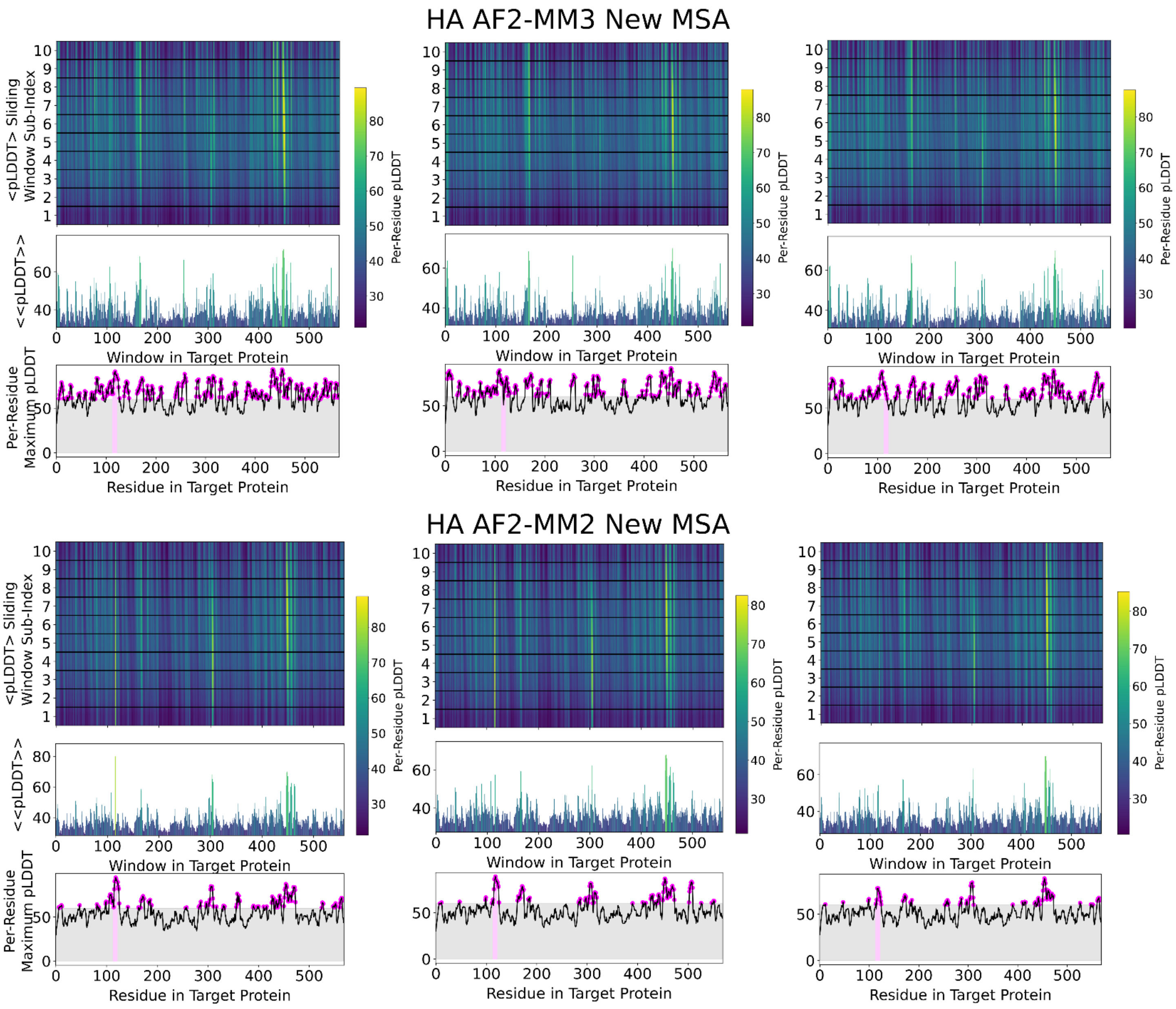

Supplemental Figure 10: HA comparison of epitope identification accuracy, comparing model types. A comparison of the differing AlphaFold2 models with the Myc system (multimer version 3 and 2) along with a comparison of the new MSA (most up to date version of the ColabFold MSA server uses UniRef30 (2302) amd PDB100 (220517)) vs the old MSA server (when this data was initially generated, ColabFold MSA server used UniRef30 (2202) and PDB70 (220313)). The left column is the WT scFv, the middle column is the CDR loops spliced onto the 15F11 backbone, and the right column is the CDR loops spliced onto the 2E2 backbone. For AF2-MM2 Old MSA, see Supplemental Figure 7.

bioRxiv preprint doi: https://doi.org/10.1101/2024.04.19.590298; this version posted December 20, 2024. The copyright holder for this preprint (which was not certified by peer review) is the author/funder, who has granted bioRxiv a license to display the preprint in perpetuity. It is made available under aCC-BY 4.0 International license.

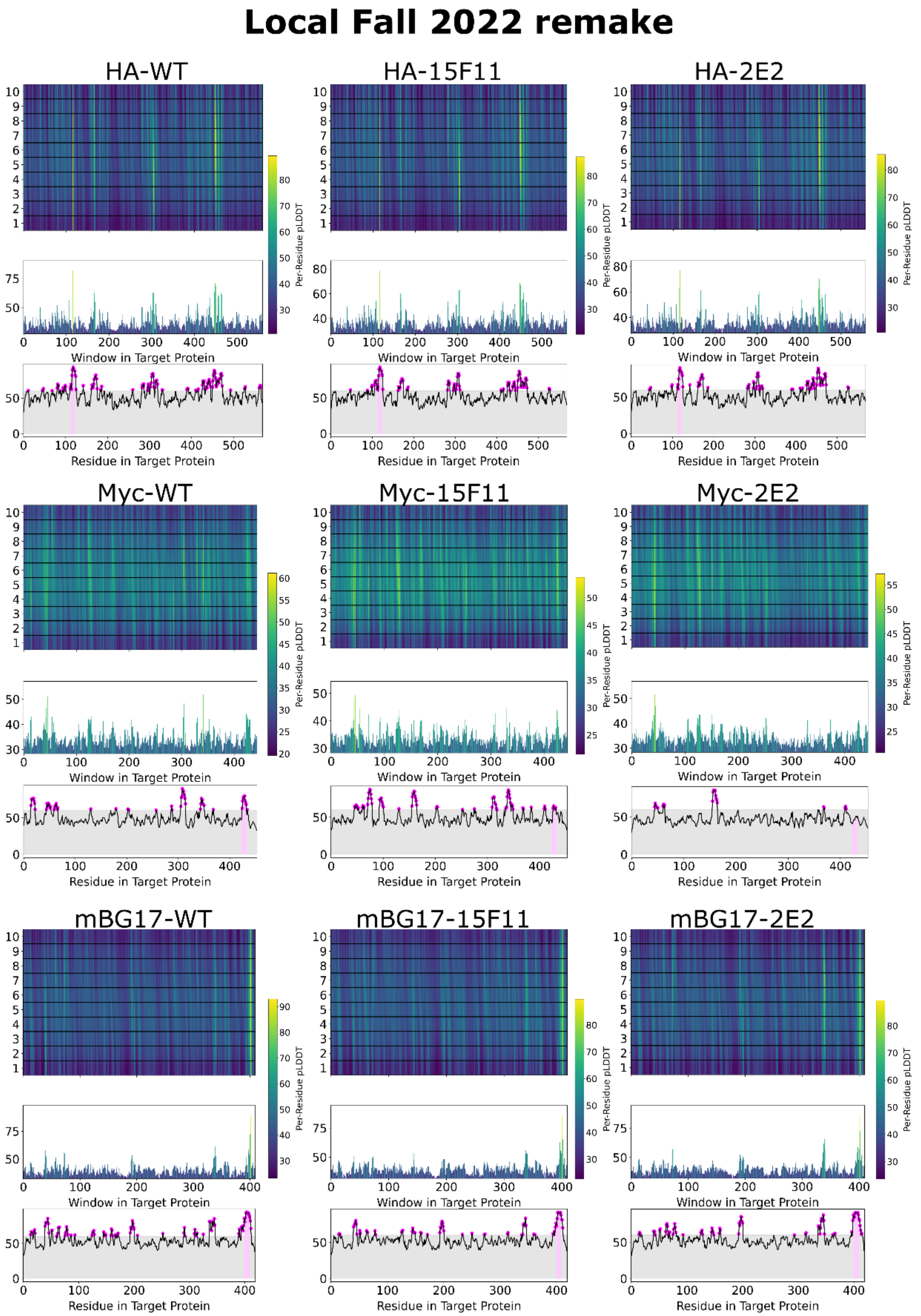

Supplemental Figure 11: Local remake of the databases used by the MMSEQS server. Databases were downloaded (UniRef30 (2202) and PDB70 (220313)) and were queried locally to produced MSA's for testing. These runs all were done with the multimer version 2 model of Alphafold 2. The left column is the WT scFv, the middle column is the CDR loops spliced onto the 15F11 backbone, and the right column is the CDR loops spliced onto the 2E2 backbone. The first row is the HA system, the second row is the Myc system, and the final row is the mBG17 system.

bioRxiv preprint doi: https://doi.org/10.1101/2024.04.19.590298; this version posted December 20, 2024. The copyright holder for this preprint (which was not certified by peer review) is the author/funder, who has granted bioRxiv a license to display the preprint in perpetuity. It is made available under aCC-BY 4.0 International license.

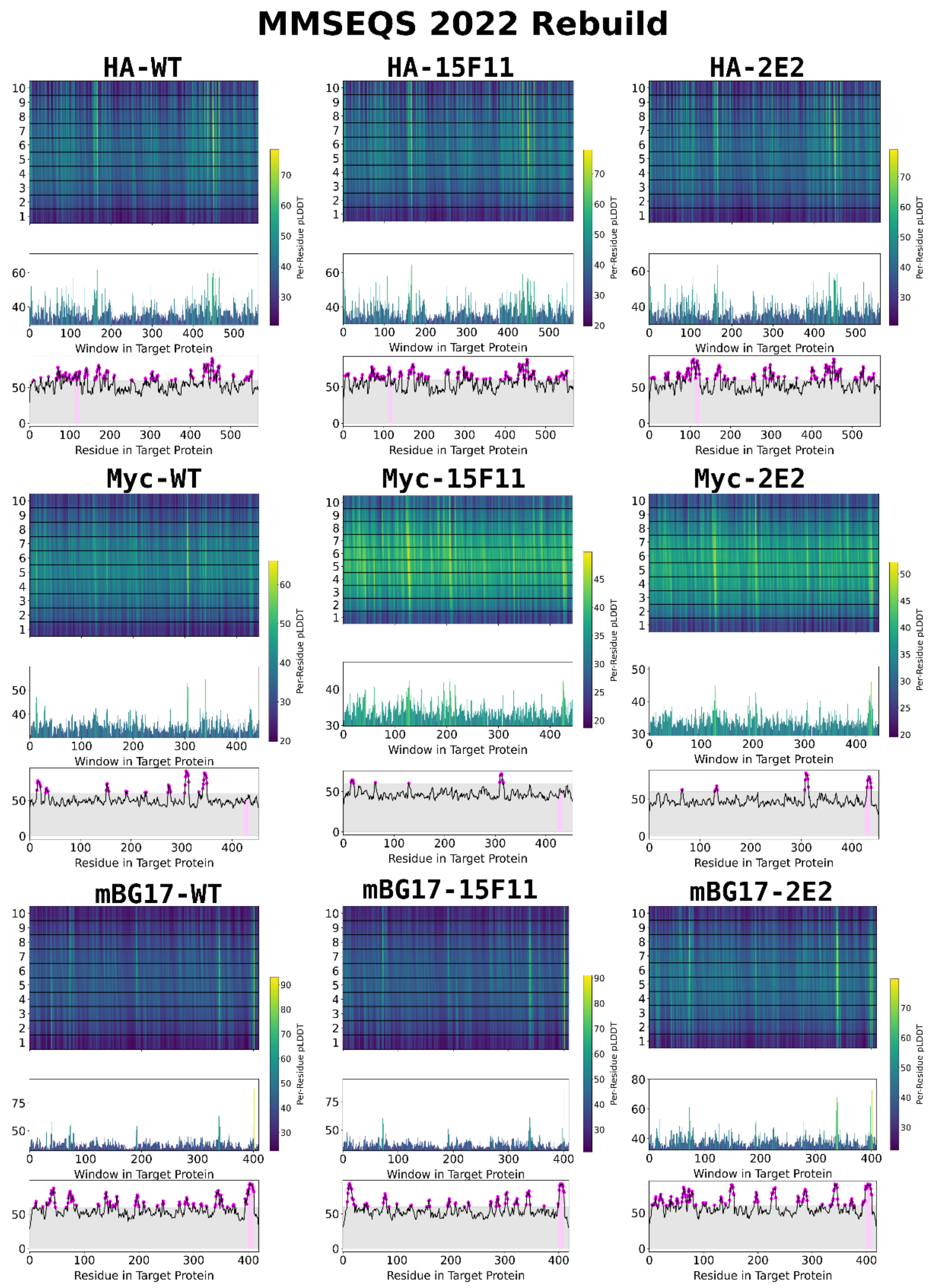

Supplemental Figure 12: Server remake of the MMSEQS databases. The databases were rebuilt by the MMSEQS team UniRef30 (2202) and PDB70 (220313)) on the Colabfold MSA server and were queried produced MSA's for testing. These runs all were done with the multimer version 2 model of Alphafold 2. The left column is the WT scFv, the middle column is the CDR loops spliced onto the 15F11 backbone, and the right column is the CDR loops spliced onto the 2E2 backbone. The first row is the HA system, the second row is the Myc system, and the final row is the mBG17 system.

bioRxiv preprint doi: https://doi.org/10.1101/2024.04.19.590298; this version posted December 20, 2024. The copyright holder for this preprint (which was not certified by peer review) is the author/funder, who has granted bioRxiv a license to display the preprint in perpetuity. It is made available under aCC-BY 4.0 International license.

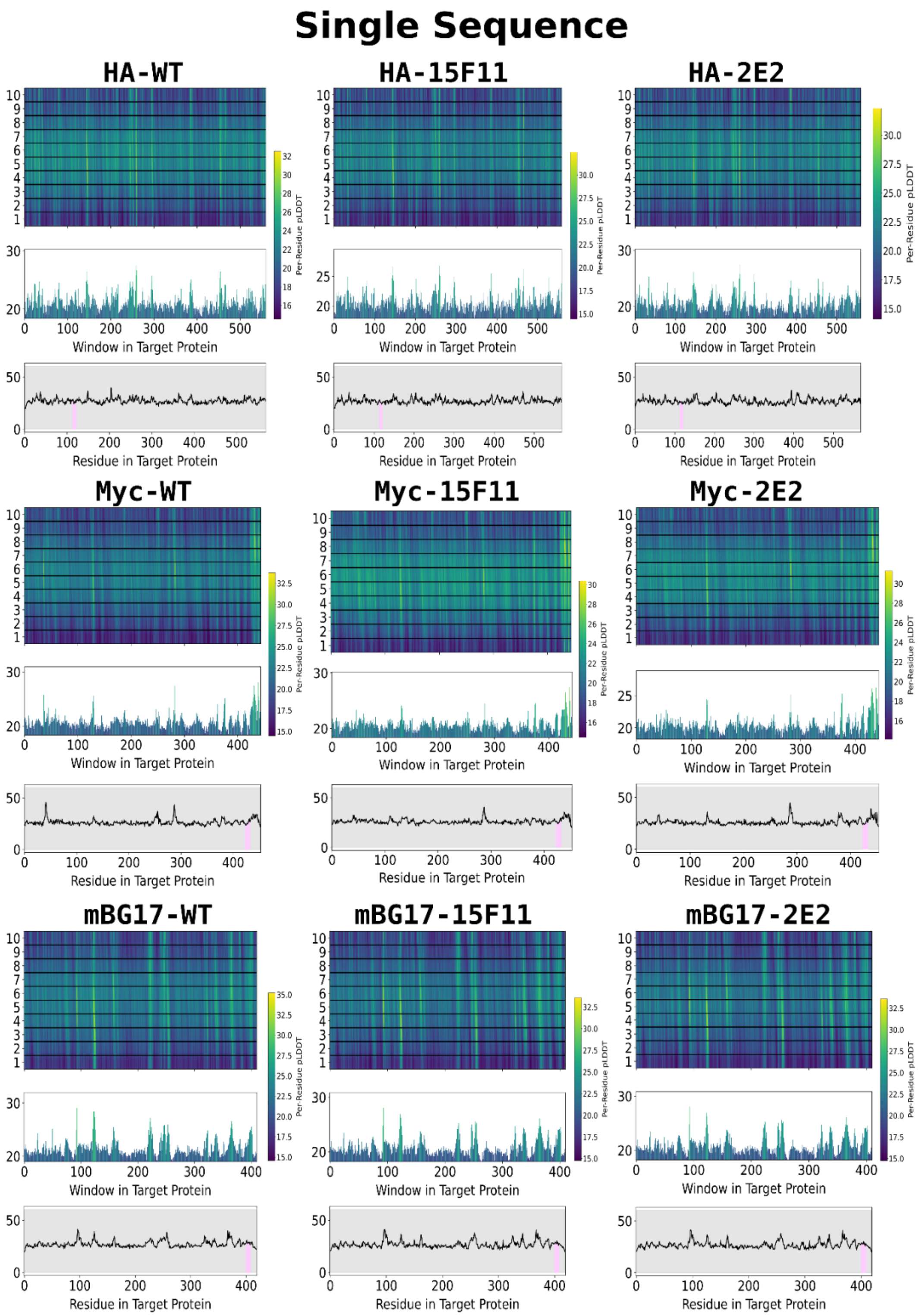

Supplemental Figure 13: Single Sequence mode (no MSA's) of epitope prediction with AF2. These runs all were done with the multimer version 2 model of Alphafold 2 in single sequence mode (i.e. no MSA was used) as a negative control, to highlight the importance of a quality MSA. The left column is the WT scFv, the middle column is the CDR loops spliced onto the 15F11 backbone, and the right column is the CDR loops spliced onto the 2E2 backbone. The first row is the HA system, the second row is the Myc system, and the final row is the mBG17 system.

bioRxiv preprint doi: https://doi.org/10.1101/2024.04.19.590298; this version posted December 20, 2024. The copyright holder for this preprint (which was not certified by peer review) is the author/funder, who has granted bioRxiv a license to display the preprint in perpetuity. It is made available under aCC-BY 4.0 International license.

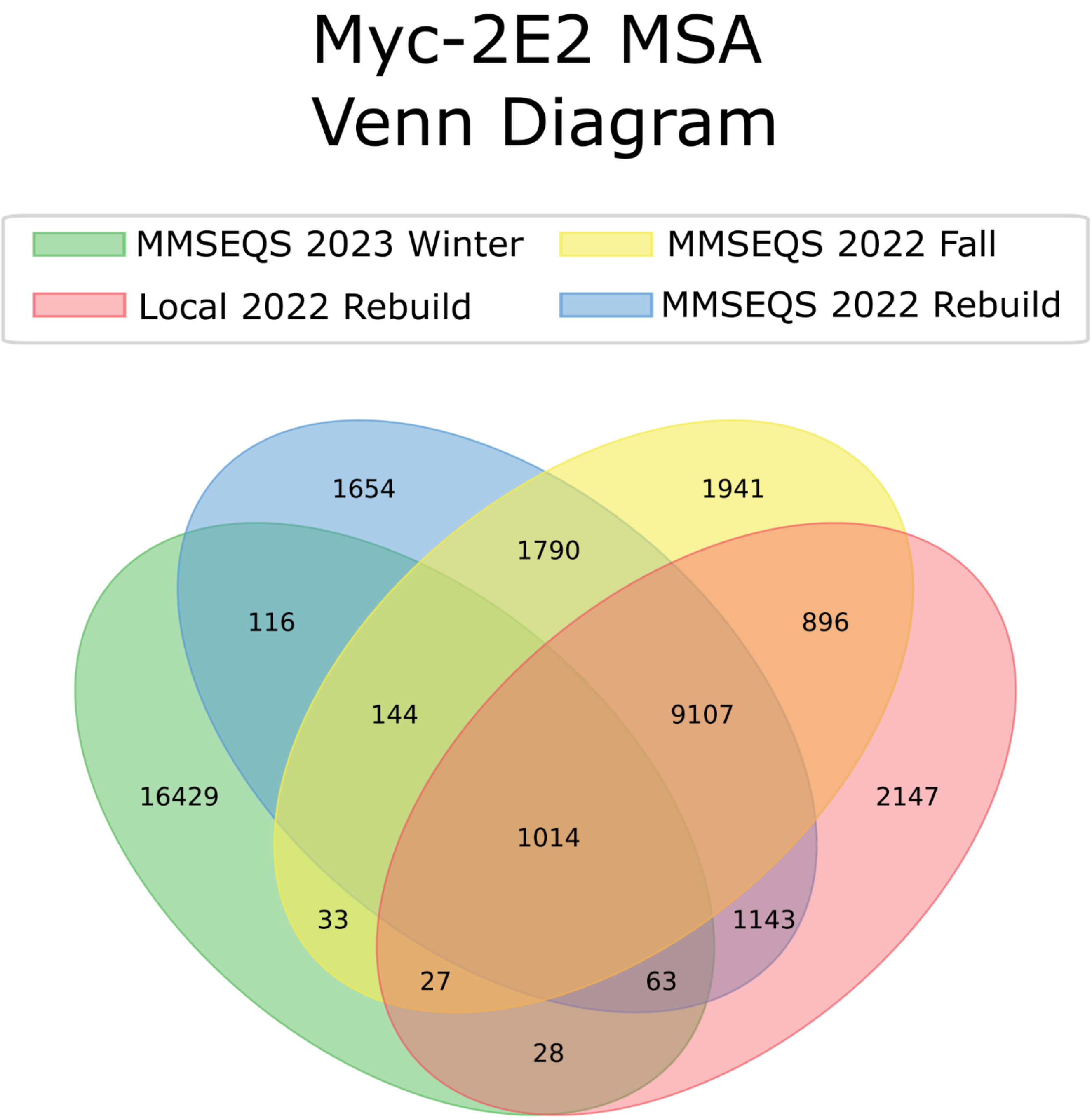

Supplemental Figure 14: MSA overlap between the 4 generation methods. Here we highlight the number of unique entries that are shared amongst all of the MSA methods, those being: **1)** using the databases right now via colabfold (PDB30 2302 and PDB100 230517) (green) **2)** the databases after they had been accessed via colabfold and cached for repeated use (UniRef30 (2202) and PDB70 (220313)) (yellow), **3)** downloading the databases locally (UniRef30 (2202) and PDB70 (220313)) and attempting to create the MSAs ourselves (red), and **4)** querying the databases after the MMSEQS team rebuilt them for our use via colabfold (UniRef30 (2202) and PDB70 (220313)) (blue).

bioRxiv preprint doi: https://doi.org/10.1101/2024.04.19.590298; this version posted December 20, 2024. The copyright holder for this preprint (which was not certified by peer review) is the author/funder, who has granted bioRxiv a license to display the preprint in perpetuity. It is made available under aCC-BY 4.0 International license.

		MMSEQS 2022 Fall	Local 2022 Rebuild	MMSEQS 2022 Rebuild	MMSEQS 2023 Winter	Single Sequence
**HA**	WT	M	✓	-	✓	-
	15F11	✓	✓	-	M	-
	2E2	M	✓	-	-	-
**Myc**	WT	M	✓	-	-	-
	15F11	✓	-	-	-	-
	2E2	✓	-	✓	✓	-
**mBG17**	WT	✓	✓	✓	✓	-
	15F11	✓	✓	✓	✓	-
	2E2	✓	✓	✓	✓	-

Supplemental Figure 15: Comparison of how well each MSA generation scheme accurately identified the experimentally derived epitope within the top 5 epitope sequences. A green checkmark shows that it was found by both the consensus model and the top single model, a yellow "M" means the simple max method correctly identified the experimental epitope in the top 5 epitopes, and the red dash means both methods failed. The consensus model did not identify the epitope correctly when the simple max method failed to. The colored background behind the titles is the same color as Supplemental Figure 14 to help guide the eye.
